# Induction of larval settlement in crown-of-thorns starfish is not mediated by conspecific cues

**DOI:** 10.1038/s41598-023-44422-x

**Published:** 2023-10-10

**Authors:** Peter C. Doll, Sven Uthicke, Ciemon F. Caballes, Frances Patel, Maria del C. Gomez Cabrera, Bethan J. Lang, Morgan S. Pratchett

**Affiliations:** 1 Australian Research Council (ARC) Centre of Excellence for Coral Reef Studies, James Cook University, Townsville, QLD 4811 Australia; 2 College of Science and Engineering, James Cook University, Townsville, QLD 4811 Australia; 3 Australian Institute of Marine Science, Townsville, QLD 4810 Australia; 4 University of Guam – Marine Laboratory, Mangilao, GU 96923 USA

**Keywords:** Marine biology, Ecology

## Abstract

Population irruptions of crown-of-thorns starfish (COTS; *Acanthaster* spp.) remain a major cause of coral reef degradation throughout the Pacific and Indian Oceans and are inherently modulated by larval settlement and recruitment success. Gregarious larval settlement, as exhibited by many other ecologically important marine invertebrates, can catalyse population growth and replenishment. However, whether conspecific cues induce or influence the settlement of COTS larvae remains a critical information gap. This experimental study examined the induction of COTS settlement in response to a range of conspecific cues associated with early- and late-stage herbivorous juveniles, corallivorous juveniles and adults. Competent COTS larvae were generally not induced to settle by the presence of conspecifics or cues associated with conspecifics, while the settlement success of COTS in the presence of coralline algae was not inhibited or enhanced by adding conspecific conditioned seawater. Rather than being reinforced by gregarious settlement, the recruitment of COTS populations appears dependent on associative settlement cues (i.e., coralline algae and/or associated microbial communities) signalling suitable benthic habitat.

## Introduction

Cues signaling the presence of conspecific animals can be an effective indicator of habitat suitability, which can be used as an adaptive strategy to form aggregations that confer density-dependent benefits to members^1–4^. The ability of conspecifics to respond to these cues appears particularly important at major ontogenetic transition points when naïve animals arrive in unfamiliar environments^5^. While pertinent information may be gathered from various inputs, including tactile stimuli, it is often chemical signals (i.e., semiochemicals) that elicit salient developmental, physiological and behavioural responses by conspecific individuals (reviewed by^6^).

Conspecific cues have been implicated in the larval settlement behaviour of many marine invertebrates with bipartite life histories (reviewed by^7,8^), including ecologically important echinoderms^9–13^. During their planktonic-benthic transition phase (i.e., larval settlement), conspecific cue-mediated (or gregarious) settlement presents an avenue for planktonic larvae to select favourable benthic habitat and thereby enhance survival probability at and after this ontogenetic boundary^14^. In most cases, the settlement response (including metamorphosis) is induced by surface-bound and/or waterborne chemical cues, which derive from conspecific juveniles or adults^7^. For example, sea urchin and sand dollar larvae settle in the isolated presence of conspecific juveniles or adults, their faeces, and conspecific-conditioned water or surfaces^12,13^. Because this gregarious settlement behaviour commonly manifests in strongly patterned recruitment^15,16^, it can have profound effects on the oftentimes irruptive population dynamics of echinoderms and other benthic marine invertebrates^8,14^.

In the case of the corallivorous crown-of-thorns starfish (COTS), conspecific cue-mediated settlement would play a key role in the localised proliferation of COTS on coral reefs. Gregarious recruitment mechanisms as documented for other marine invertebrates could result in positive feedback loops that reinforce population replenishment and rapid population growth of COTS^17^, exacerbating their pervasive threat to the structure and function of coral reef ecosystems. While the ability of adult COTS to respond to waterborne chemical cues derived from conspecifics is well established^18–21^, evidence of cue-mediated behaviour prior or during larval settlement is highly fragmented. Attraction of COTS larvae to conspecific cues was initially hypothesised based on the relatively high abundance of late-stage juveniles at a reef within the adult outbreak area^22^. This hypothesis was only recently corroborated in a static choice chamber experiment, which documented horizontal movement of late-stage COTS larvae towards adult conspecifics^23^ and has since been considered as an avenue for COTS population replenishment^24–26^. However, whether the ecologically fundamental process of larval settlement is induced by conspecifics remains a critical information gap for COTS^27^.

The main objective of this study was thus to test whether COTS larvae settle in response to the presence of conspecifics and to cues associated with conspecifics. Considering the diversity of gregarious cues known to induce marine invertebrate settlement (reviewed by^7,8^), we examined the specific nature of the potential gregarious cue by assessing surface-bound and waterborne cues associated with early- and late-stage herbivorous juveniles, corallivorous juveniles and adults, representing the different life history-stages that COTS larvae may encounter in the natural environment. Given that COTS settlement is already known to be induced by a wide range of coralline algae-associated cues^28^, this study also assessed the interactions between conspecific cues and coralline algae-mediated settlement induction. Importantly, filling these gaps in our understanding of the chemical cues that trigger COTS settlement could improve the ecological underpinning of current culling efforts and/or aid the development of innovative population control methods using semiochemicals^21^.

## Methods

### Spawning and larval rearing

Adult western Pacific crown-of-thorns starfish (COTS, *Acanthaster* cf. *solaris*) were collected from mid-shelf reefs in the central Great Barrier Reef (GBR) and held in flow-through unfiltered seawater tanks (26.5 ± 0.5 °C) at the Australian Institute of Marine Science National Sea Simulator. COTS were sexed upon collection using a hypodermic needle^29^, with males and females transported and kept separately in the flow-through tanks. The water temperature used throughout the subsequent rearing and experiment processes of both experiments (28 ± 0.5 °C) represents summer temperatures (COTS reproductive season) in the collection area.

COTS were spawned on November 24th, 2021, for Experiment 1 (juvenile conspecific cues) and November 7th, 2022, for Experiment 2 (adult conspecific cues). For each spawning, we obtained a small number of ovary lobes from six female COTS by making small incisions at the proximal ends of their arms. The ovary lobes were then rinsed with filtered seawater (fsw) through a 500 μm mesh to remove any loose eggs. To induce the maturation and release of the remaining eggs, the ovary lobes were placed into beakers containing 200 mL fsw with 1-methyladenine (treatment concentration: 10^–5^ M) for 60 min^30^. Approximately 20 min prior to the completion of the maturation step, we obtained sperm from the arms of six male COTS and 2 μL of dry sperm from each male was mixed in 15 mL fsw. After the eggs were rinsed through a 500 μm mesh to remove any unshed eggs or connective tissues, the number of eggs per mL was counted in the stock solution. We added 1 mL of the sperm stock solution to the egg stock solution and observed successful fertilisation of more than 80% using a stereo microscope. The fertilised embryos were then divided between two 70 L vats with low air line setting at a density of approximately 10 embryos ml^-1^.

Twenty-four hours post-fertilisation, 100% water exchanges were conducted to remove any undeveloped embryos and dead larvae. At 48 h post-fertilisation, the water exchange process was repeated to concentrate healthy larvae and larval stages were scored using stereo microscopes. We then moved the larvae into 16 L flow-through culture cones, stocked at approximately 1 larvae mL^−1^. At 5 days post-fertilisation, larvae started feeding on *Dunaliella* sp. (CSIRO CS-353; Australian National Algae Culture Collection Strain List 2022) and *Isochrysis* sp. (CSIRO CS-177) stock cultures. All rearing tanks were supplied with the algal feeds (1200–1500 cells per ml) via automatically dosed treatment tanks with controlled chlorophyll concentrations^31^. Full water changes were carried out three times a week, whereby healthy larvae were siphoned into holding buckets while rearing cones, tubes and air lines were thoroughly cleaned. We regularly examined larval development using stereo microscopes throughout the rearing process and determined larvae competent to settle once they reached the late-brachiolaria stage with a well-developed rudiment. Metamorphic competency was further corroborated by 24 h trials using a coralline alga known to induce settlement (*Lithothamnion* cf. *proliferum*) and, for both experimental larval batches, competency was confirmed at 14 days post-fertilization (December 9th 2021 and November 21st 2022). Because multiple culture cones were required for the experiments, larvae from the different cones were thoroughly mixed before the randomized allocation to treatments.

### Experimental treatments

Experiment 1 (juvenile conspecific cues) and 2 (adult conspecific cues) consisted of nine and five experimental treatments, respectively (Table 1), including positive (treatments 1.7 and 2.4^28^) and negative controls (treatments 1.9 and 2.5). To obtain 1-month old juveniles for Experiment 1 (treatments 1.1 and 1.4), COTS were spawned on November 9th 2021, and larvae were reared following the aforementioned protocol. Settlement was facilitated using the coralline alga *Lithothamnion* cf. *proliferum* and post-metamorphic juveniles were raised on a mixed coralline algae diet at the National Sea Simulator for 2 weeks. To obtain 1-year old juveniles (treatments 1.2, 1.3, 1.5, 1.6 and 1.8), COTS larvae were reared following the same protocol and settled in early December 2020. The herbivorous juveniles were raised on a mixed coralline algae diet for 12 months and a subset of this juvenile cohort was transitioned to a coral diet (*Acropora* spp.) six weeks prior to experiment commencement (treatments 1.3 and 1.6). At the start of the experiment, the mean sizes of the 1-year old herbivorous and corallivorous juveniles were 0.84 cm (± 0.04 se) and 1.19 cm (± 0.04 se), respectively. The conditioned fsw treatments (1.4–1.6) were obtained by placing five individuals from each juvenile cohort (1.1–1.3) in glass aquaria filled with 810 mL fsw for 48 h. Pieces of *Lithothamnion* cf. *proliferum* (treatments 1.7, 1.8 and 2.4) were identified based on morpho-anatomical features and molecular sequencing^32^ and cut into replicate 0.5 × 0.5 cm chips prior to experiment commencement.

**Table 1:** Treatments used in settlement assays with crown-of-thorns starfish larvae (*n* = 12 independent and randomised wells for each treatment).

Treatment	Description
Experiment 1	
1.1 1-m (alg)	1-month old herbivorous juvenile (coralline algae spp.)
1.2 1-y (alg)	1-year old herbivorous juvenile (coralline algae spp.)
1.3 1-y (cor)	1 year-old corallivorous juvenile (*Acropora* spp.)
1.4 csw 1-m (alg)	fsw conditioned with 1-month old herbivorous juveniles (1.1)
1.5 csw 1-y (alg)	fsw conditioned with 1-year old herbivorous juveniles (1.2)
1.6 csw 1-y (cor)	fsw conditioned with 1-year old corallivorous juveniles (1.3)
1.7 *L. pro*	0.5 × 0.5 cm live coralline algae chip (*Lithothamnion* cf. *proliferum*) (positive control)
1.8 *L. pro* + csw	Herbivorous 1-y-o juvenile conditioned fsw (1.5) added to coralline algae chip (1.7)
1.9 fsw	Filtered seawater (negative control)
Experiment 2	
2.1 tube foot	Adult tube foot obtained from single 20 cm Ø male
2.2 spine	Adult spine piece (1.5 cm length) obtained from single 20 cm Ø male
2.3 csw	Conditioned seawater from 1200 L flow-through tank containing 12 males (15–35 cm Ø)
2.4 *L. pro*	0.5 × 0.5 cm live coralline algae chip (*Lithothamnion* cf. *proliferum*) (positive control)
2.5 fsw	Filtered seawater (negative control)

### Settlement assays and analysis

To test whether the presence of conspecifics and conspecific cues induce COTS larvae to settle, 12 replicate settlement assays were conducted for each of the experimental treatments. Using six-well cell culture plates, we fully randomised the distribution of all replicate assays among the 108 and 60 wells for the juvenile and adult conspecific cue experiments, respectively. Upon adding 10 mL fsw and the treatments, we carefully pipetted approximately 10 competent COTS larvae to each well. Settlement success was scored 24 h after the larvae were introduced using stereo microscopes. For each replicate assay, we recorded the number of swimming late-brachiolaria larvae and the number of individuals which attached to the treatment or well bottom and commenced or completed metamorphic transformation into a juvenile with radial symmetry (= settled).

Statistical analysis was performed using R software (v. 4.1.3^33^). Mean settlement success for each treatment was calculated and plotted based on the settlement rates obtained from replicate assays (*n* = 12 per treatment), considering the proportion of swimming larvae and settled postlarvae. To determine whether settlement success in the presence of coralline algae (treatment 1.7) may be inhibited or enhanced by adding conspecific conditioned seawater (treatment 1.8), a two-sample *t* test was performed for treatments 1.7 and 1.8 using the *stats* package^33^. Figures were generated using the *ggplot2* package in R^34^.

## Results and discussion

**Figure 1:**
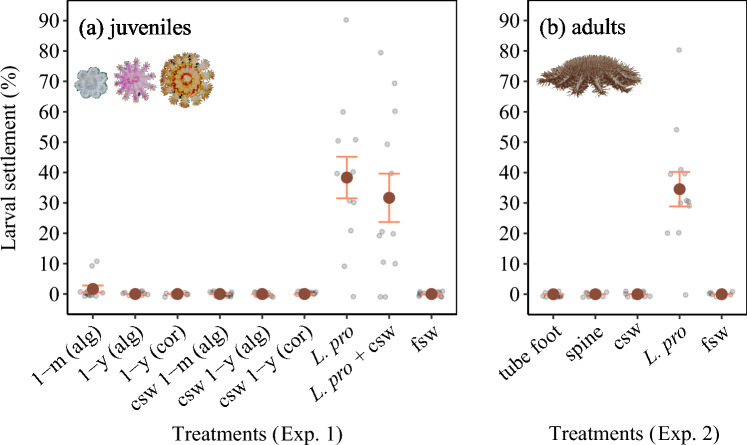
Settlement success of crown-of-thorns starfish larvae (means ± standard error, *n* = 12) for each treatment, calculated based on replicate assay settlement rates (grey points). Descriptions of the experimental treatments are listed in Table 1.

Establishing whether COTS larvae settle gregariously advances our understanding of ecological mechanisms that drive population growth and replenishment in this keystone coral predator. Here, we found no evidence of COTS settlement induction in the presence of conspecifics or by cues associated with conspecifics (Fig. 1). While two individuals had commenced metamorphosis in the presence of 1-month-old herbivorous juveniles (1.67% settlement ± 1.12 se; *n* = 120 larvae), this likely denotes spontaneous settlement in the absence of a suitable settlement cue, which has previously been observed in small proportions of late-stage COTS larvae^35,36^. No larvae settled across all other juvenile or adult conspecific treatments (Fig. 1), which is in direct contrast to the hypothesis suggesting that COTS settlement may be mediated by conspecific cues^22,24–26^. This hypothesis was largely based on the field observation of relatively high juvenile abundance on a reef within an adult population outbreak area^22^ and documented larval movement towards adult conspecifics in a static choice chamber experiment^23^. While some degree of conspecific chemo-attraction of COTS larvae towards conspecifics is conceivable^21^ and may influence the movement of planktonic larvae prior to settlement, our results suggest that COTS settlement induction and concomitant spatiotemporal settlement patterns must be largely governed by other environmental inputs, such as coralline algae-associated cues^28^.

The absence of conspecific-cue mediated settlement in COTS stands in contrast to the conspicuous mechanisms and patterns of gregarious settlement in other echinoderms (reviewed by^14^) and many ecologically important marine invertebrate taxa^37–41^. Chemical cues released by conspecifics have been shown to induce larval settlement responses in sea urchins^13,42^, sand dollars^9,11,43,44^, sea cucumbers^10,45^, brittle stars^46–48^, and feather stars^49–51^. However, the identification of specific chemical compounds triggering this behaviour has proven elusive in most cases^14^, with the exception of a small peptide responsible for settlement induction in the sand dollar *Dendraster excentricus*^9,11,43^. Independent of this gregariousness, plasticity in settlement behaviour is generally quite prevalent among echinoderms^8,14^, contrasting the seemingly high cue specificity displayed by COTS larvae^28,52^.

Notably, the results of this study also provided no evidence that the addition of conspecific conditioned seawater inhibits or enhances settlement success in the presence of a known settlement cue, the coralline alga *Lithothamnion* cf. *proliferum* (Fig. 1; two-sample *t* test, *t* = 0.64, df = 22, *P* = 0.532). Mean settlement success for wells containing *L*. cf. *proliferum* (38.33% ± 6.83 se) and the combination of *L*. cf. *proliferum* and seawater conditioned with conspecific juveniles (31.67% ± 7.96 se) was comparably high and similar to previously reported COTS settlement rates in the presence of this coralline alga^28^. Planktonic COTS larvae are unlikely to encounter isolated environmental stimuli in the field but are instead often exposed to a suite of waterborne chemical cues with the potential to promote or inhibit settlement^14^. On coral reefs, the same rubble environments in which herbivorous COTS juveniles are usually observed also provide large amounts of the food source (i.e., coralline algae) required for this life-history stage^25,53,54^. Our results indicate that the selection of rubble habitats by settlement-stage COTS larvae is not affected by the presence of conspecific juveniles inhabiting these reef environments.

Although the prevalence of gregariousness in benthic marine invertebrate communities indicates that the benefits may outweigh the costs^8^, the lack of gregarious settlement behaviour displayed in this study is not necessarily disadvantageous for COTS, at least at the individual organism level. There are clear trade-offs between potential advantages (e.g., favourable habitat, including food availability) and disadvantages (e.g., competition for food) of this phenomenon for members of resulting aggregations^7,8,55,56^. On balance, the presence of conspecifics usually signals suitable environmental conditions, however, planktonic larvae may obtain some of the same benefits by responding to associative cues, originating from heterospecific organisms such as juvenile food sources^57^, without the potential costs associated with conspecific aggregations. Considering the relatively narrow ecological niche of COTS during their herbivorous juvenile stage^53^, settlement in the proximity of juveniles would likely be maladaptive, since limited distribution of individuals and concomitant food competition for coralline algae may inhibit their early post-settlement growth and fitness^8^. It is not surprising that settling COTS larvae also appear insensitive to the presence of larger coral-feeding individuals, given marked differences in their diet and habitat requirements^58^.

The relative importance and influence of conspecific settlement cues among echinoderms and other marine invertebrates is clearly taxon-specific and warrants further research for ecologically important groups. Our results suggest that COTS larvae require associative cues originating from coralline algae and/or biofilms^28,52^, rather than gregarious cues, for the successful transition to their benthic juvenile stage. The associative settlement of planktonic larvae (in response to heterospecific plant or animal species^57^), especially biofilm- or bacteria-induced settlement, is a widespread mechanism among marine invertebrates^59^, including echinoderms^14^. While the specificity and relative importance of these cues remains poorly understood for most taxa, some bacteria and compounds are particularly important to the successful induction of marine invertebrate settlement^60–62^. Likewise, cues derived from specific algae and/or bacteria appear necessary for COTS larvae to undergo the cascade of behavioral events associated with settlement^14^, although prior to settlement, COTS larvae may also be attracted to chemical cues released by conspecifics and move towards them during their planktonic phase^23^. In any case, the absence of salient gregarious settlement behaviour as we know it from other benthic marine invertebrates represents good news for coral reefs featuring high adult COTS densities, because it diminishes the likelihood of high self-recruitment and positive feedback loops that reinforce population growth and replenishment.

## Data Availability

The data generated as part of this study are available from Research Data JCU (https://doi.org/10.25903/r8hn-tn02).
